# A novel clinical biomarker-based Physiology Healthy Aging Index and risk of all-cause and cause-specific mortality: A 20-year prospective cohort study

**DOI:** 10.1007/s11357-025-02025-6

**Published:** 2025-12-06

**Authors:** Jieun Lyu, Ji-Yun Hwang, Joong-Yeon Lim, Yoon Jung Park

**Affiliations:** 1 Division of Population Health Research, Department of Precision Medicine, National Institute of Health, Cheongju, 28159 Republic of Korea; 2 Department of Nutritional Science and Food Management, Ewha Womans University, Seoul, 03760 Republic of Korea; 3 Major of Foodservice Management and Nutrition, Sangmyung University, Seoul, 03016 Republic of Korea; 4 Graduate Program in System Health Science & Engineering, Ewha Womans University, Seoul, 03760 Republic of Korea

**Keywords:** Physiological Healthy Aging Index, Aging, Mortality, Biomarkers, Cohort study, Korean population

## Abstract

Population aging is accelerating worldwide, with 16% projected to be aged ≥ 65 years by 2050. A practical index reflecting overall aging status is needed for population-based research, as existing indices often require specialised or cognitive assessments. We developed a Physiological Healthy Aging Index (PHAI) using accessible biomarkers and evaluated its association with mortality in Korean adults. A total of 6398 participants aged ≥ 40 years from the Korean Genome and Epidemiology Study (KoGES) Ansan-Ansung cohort followed up for an average duration of 16.5 years (2001–2022). The PHAI, based on systolic blood pressure, fasting blood glucose, serum creatinine, forced vital capacity, and C-reactive protein levels, was scored 0–10, with higher scores indicating healthier aging. Mortality risks across quartiles were estimated using Cox proportional hazard models. Long-term changes were classified as accelerated (decreased scores), stable (unchanged scores), or resilient (increased scores). During 105,597 person-years, 934 deaths occurred (778 age-related, 353 cancer-related, and 184 cardiovascular-related). Higher PHAI quartiles were linked with significantly lower mortality risk versus Q1. Fully adjusted hazard ratios (95% CIs) for all-cause mortality were 0.82 (0.69–0.98) for Q2, 0.50 (0.42–0.60) for Q3, and 0.51 (0.41–0.63) for Q4 (*P* for trend < 0.001). Similar associations were observed for age-related mortality (HR 0.51, 95% CI 0.40–0.64 for Q4 vs. Q1), cancer (HR 0.66, 95% CI 0.48–0.92), and cardiovascular mortality (HR 0.24, 95% CI 0.13–0.44). Resilient agers had much lower all-cause mortality than accelerated agers (HR 0.21, 95% CI 0.16–0.28), with stable agers also at reduced risk (HR 0.65, 95% CI 0.54–0.77). Higher scores also correlated with a lower cognitive impairment risk. The PHAI is a simple, robust predictor of mortality outcomes, supporting its use as a practical tool for assessing physiological aging in public health and clinical settings.

**Graphical Abstract:**

**Figure:**
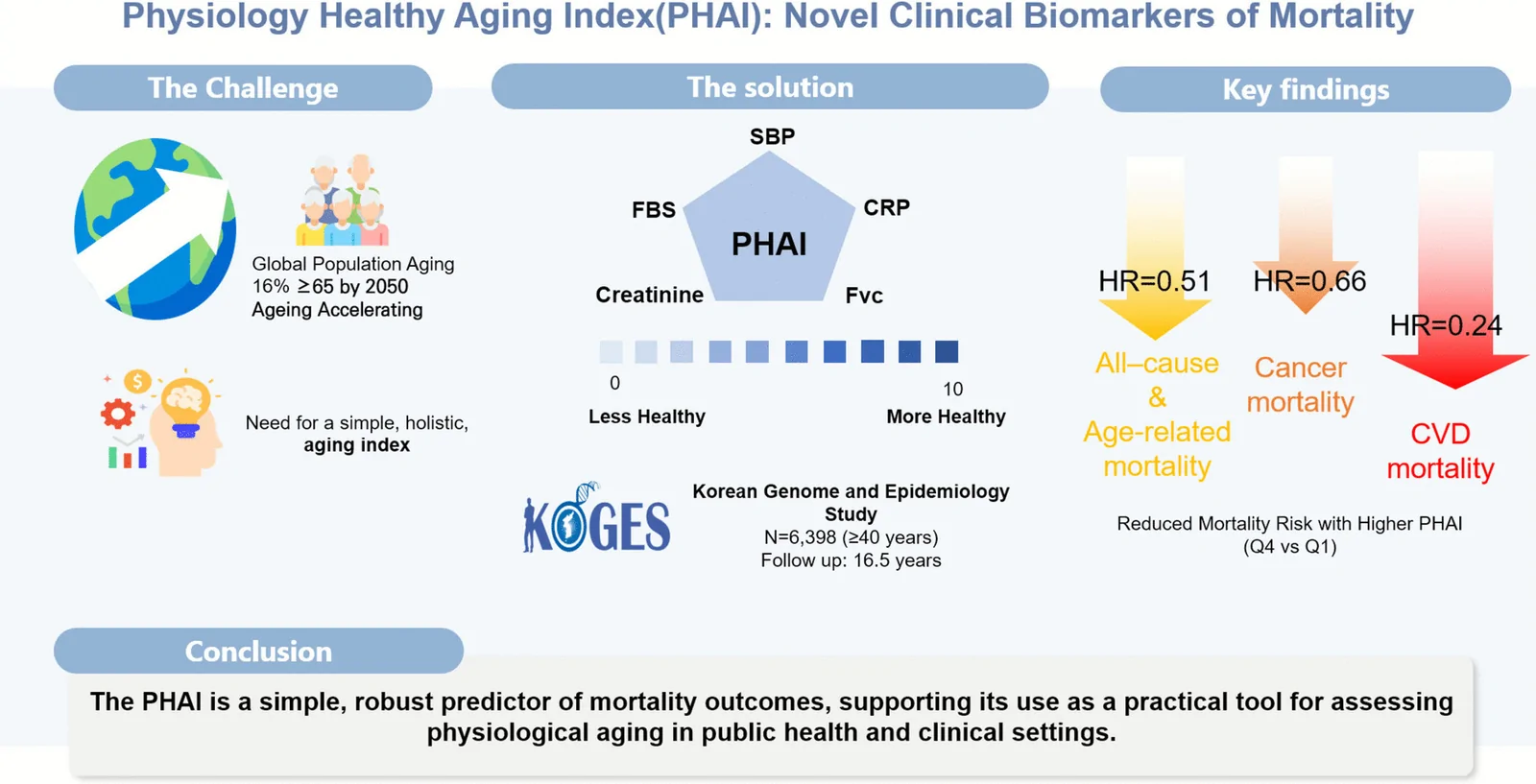

**Supplementary Information:**

The online version contains supplementary material available at https://doi.org/10.1007/s11357-025-02025-6.

## Introduction

The global population of older adults is increasing rapidly. In 2022, approximately 771 million people aged 65 and older constituted about 10% of the global population, with projections indicating an increase to 16% by 2050 and 24% by 2100 [1]. The phenomenon of population aging is especially significant in Asia. By 2050, Japan, Hong Kong, and South Korea are estimated to have nearly 40% of their populations aged 65 and older, the highest rate globally [2, 3]. In Korea, individuals aged ≥ 65 years will account for 19.2% of the total population in 2024, with projections indicating an increase to 30% by 2036 and 40% by 2050 [4].

Aging is a complex biological phenomenon characterised by a progressive decline in physiological integrity, impaired organ function, heightened disease susceptibility, and ultimately death. With the global rise of an aging population, research on healthy aging has become a significant societal concern [5]. ‘Healthy aging’ refers to maintaining functional ability and overall well-being in advancing age. It enhances individual quality of life, reduces the incidence of age-related chronic diseases, and maintains independence [6, 7]. Due to their diverse lifestyles and physiological responses, individuals age differently, leading to discrepancies in biological aging that do not necessarily correspond to their chronological age. Chronological age merely reflects the passage of time, whereas biological age accurately captures an individual’s functional capacity and vulnerability to disease [8–11]. Identifying reliable biomarkers of biological age is essential for objectively assessing the healthy aging status and aging trajectories, predicting health outcomes, and evaluating the efficacy of interventions designed to enhance longevity and health span [12, 13]. They can function as crucial tools in epidemiological research, clinical practice, and public health policy by facilitating the early identification of age-related decline, stratification of aging phenotypes, and targeted preventive strategies, ultimately promoting the advancement of personalised approaches to aging.

Recent studies indicate that numerous physiological alterations transpire during the aging process, and initiatives are underway to develop health indicators related to aging based on these findings [14–18]. Newman et al. developed the Healthy Aging Index (HAI) based on five physiological markers, including carotid intima-media thickness, fasting serum glucose, pulmonary vital capacity, serum cystatin C, and white matter grade [14]. Several cohort studies have confirmed the HAI as a reliable predictor of mortality, morbidity, and disability [15–18]. However, these indicators are infrequently collected in most epidemiological cohorts, and data on cognitive function are often accessible only in specialised studies or cross-sectional cohorts, limiting their applicability in general population-based cohorts [19].

The limitation underscores the need for a simple yet robust physiological aging index that can be readily implemented in large epidemiological cohorts. In this study, we developed and validated a PHAI using accessible clinical biomarkers within the Korean Genome and Epidemiology Study (KoGES) cohort to examine its association with mortality. By substituting C-reactive protein for neurocognitive measures, the PHAI incorporates a key inflammatory dimension of aging while preserving the cardiovascular, metabolic, renal, and respiratory domains included in established indices. Chronic low-grade inflammation, or ‘inflammaging’, is a recognised hallmark of biological aging that contributes to diverse age-related pathologies [20]. This modification also enables us to test whether physiological aging indices can predict cognitive decline even in the absence of direct cognitive assessments, thereby providing evidence for shared mechanistic pathways linking systemic aging processes across organ systems.

## Methods

### Study population

This extensive prospective study utilised data from the Ansan and Ansung cohorts of the KoGES [21]. The Ansan and Ansung community-based cohort study was initiated in 2001 and enrolled 10,030 participants from an industrialised community in Ansan and a rural region in Ansung, South Korea. The participants were followed up every 2 years, and their baseline and biennial data on sociodemographic characteristics, medical history, family history, and lifestyle behaviours obtained through interviews using structured questionnaires and standardised protocols.

**Fig. 1:**
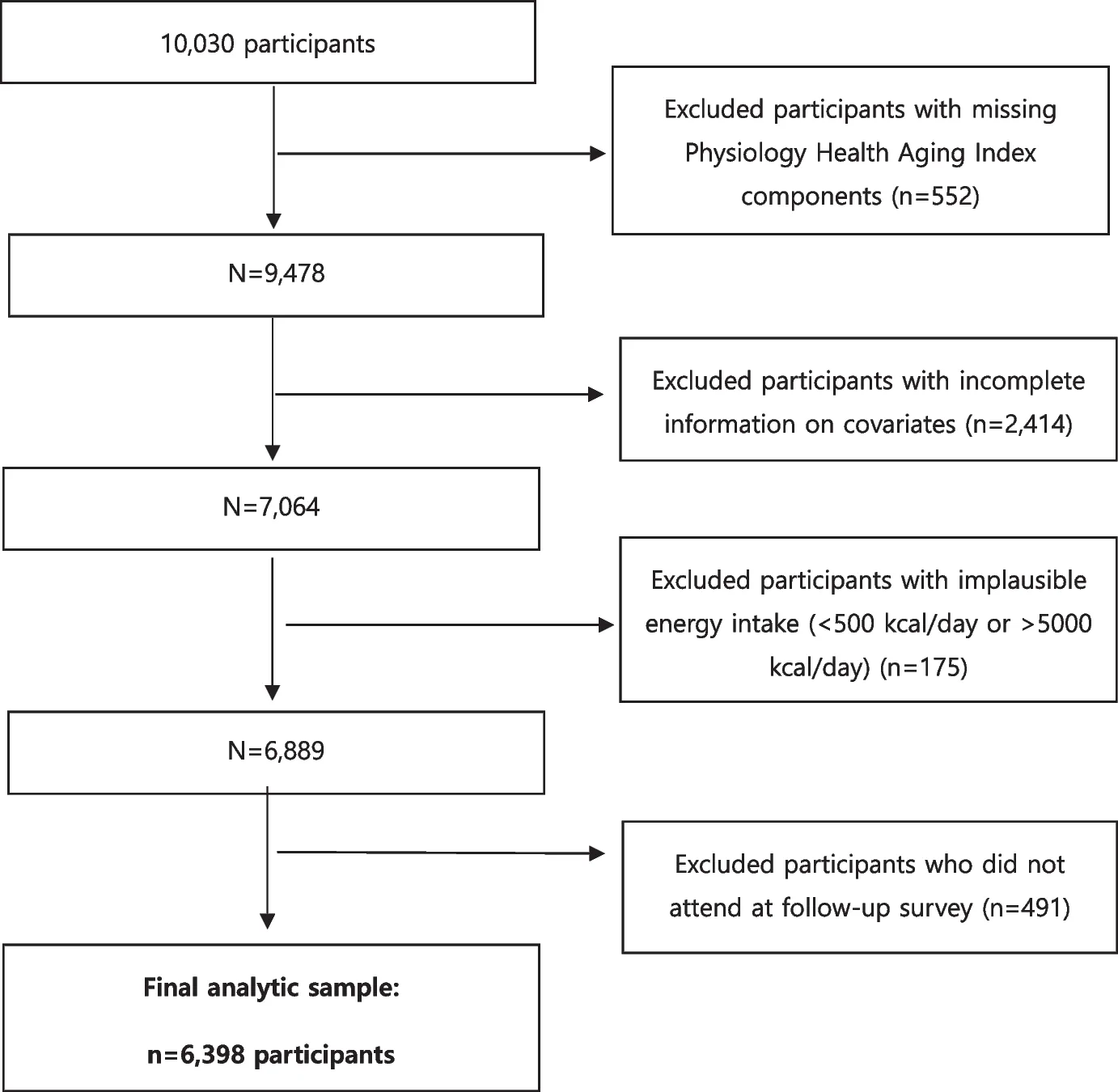
Flow diagram of the study participants. This flowchart illustrates the participant selection process and exclusion criteria applied to derive the final study population for the Physiology Healthy Aging Index analysis

The Ansan and Ansung cohort data were analysed up to 2022 in this study. From the 10,030 participants who consented to link with the national cause of death registry, we excluded individuals based on the following criteria: those with incomplete data on Physiological Healthy Aging Index components (*n* = 552), those lacking data on covariates (*n* = 2,414), and those with implausible energy intake (< 500 or > 5000 kcal/day) (*n* = 175). We excluded individuals who did not participate in any follow-up surveys (*n* = 491). Subsequent to these exclusions, 6398 participants were included in the study (Fig. 1). The Ansan and Ansung cohorts were approved by the institutional review boards (IRBs) of KoGES group collaborators and the Korea Disease Control and Prevention Agency (KDCA). This research was approved by the IRBs of the KDCA (IRB Nos. 2018–03–05-4C-A, 2022–07-07-C-A). All participants provided written informed consent.

### The physiological healthy aging index components

#### Systolic blood pressure

Anthropometric measurements were conducted by trained personnel following standardised protocols. Blood pressure was assessed twice in the arm using a standard mercury sphygmomanometer (Baumanometer, Baum Co., USA) following a period of at least 5 min of quiet seated rest for the participants. Systolic blood pressure values were defined as the average of two recorded measurements used for the analysis. The participants were categorised into three groups based on the clinical cutoff points for systolic blood pressure: 0 = ≥ 140 mmHg, 1 = 120–139 mmHg, and 2 = < 120 mmHg [22].

#### Fasting blood glucose

Trained staff performed blood tests adhering to standardised protocols. Fasting blood glucose was assessed using hexokinase analysis on Hitachi Automatic Analyser 7600 (from 2001 to August 2009), Siemens ADVIA 1650 (from September 2009 to 2010), or Siemens ADVIA 1800 (from 2010 to 2022) for all subjects. All participants fasted for 8 h. Participants were then classified into three groups based on the clinical cutoff points for fasting blood glucose: 0 = ≥ 126 mg/dL, 1 = 100–125 mg/dL, and 2 = < 100 mg/dL [23].

#### Creatinine

Creatinine levels were quantified using latex-enhanced immunoturbidimetric analysis on a Hitachi Automatic Analyser 7600 (from 2001 to August 2009), Siemens ADVIA 1650 (from September 2009 to 2010), or Siemens ADVIA 1800 (from 2010 to 2022). The results were scored into three groups according to clinical cutoff points for creatinine: 0 = ≥ 2.0 mg/dL, 1 = 1.4–2.0 mg/dL, and 2 = < 1.4 mg/dL [24].

#### Forced vital capacity

Forced vital capacity (FVC) values were calculated using a Sensormedics VMAX 2130. Participants were divided into three FVC categories based on sex-specific thresholds replicated from previous studies: 0 = < 3.2 L for men and < 2.1 L for women; 1 = 3.2–3.8 L for men and 2.1–2.6 L for women; and 2 = > 3.8 L for men and > 2.6 L for women [17].

#### C-reactive protein and high-sensitivity C-reactive protein

Previous studies indicate that C-reactive protein (CRP) and high-sensitivity CRP (hs-CRP) levels are biomarkers of aging. CRP levels were measured in the baseline survey, while hs-CRP levels were assessed in the follow-up survey. The CRP and hs-CRP levels were quantified using a turbidimetric assay method on a Hitachi Automatic Analyser 7600 (from 2001 to August 2009), Siemens ADVIA 1650 (from September 2009 to 2010), or Siemens ADVIA 1800 (from 2010 to 2022). Participants were classified into three groups according to clinical cutoff points as follows: 0 = ≥ 1 mg/dL for CRP and ≥ 3 mg/dL for hs-CRP, 1 = 0.5–1 mg/dL for CRP and 1–3 mg/dL for hs-CRP, and 2 = < 0.5 mg/dL for CRP and < 1 mg/dL for hs-CRP [25].

All aforementioned PHAI components are known risk factors for mortality and aging. The aggregate of these five scores resulted in a total PHAI score between 0 and 10, where a lower score signifies a poorer, unhealthier aging status. Supplementary Table 1 shows a summary of the cutoffs used in this study.

### Ascertainment of mortality

Participants were followed up from baseline until death or the end of the study period (December 31, 2022). The survey data were linked to the national cause-of-death registry using the participants’ registration numbers [26]. The causes of death were categorised in accordance with the Korean Standard Classification of Disease, 6th revision (KCD-6) [27], which is based on the International Statistical Classification of Diseases and Related Health Problems, 10th revision (ICD-10) [28]. The study outcomes included all-cause mortality, age-related mortality (excluding infectious and parasitic diseases, meningitis, encephalitis, and wounds), cancer mortality (C00-C97), and Cardiovascular Disease (CVD) mortality (I00-I99).

### Cognitive function assessment

Cognitive function was evaluated using the validated Korean version of the Mini-Mental State Examination (K-MMSE), a recognised instrument for screening cognitive impairment and assessing mental status in clinical and research settings [29]. The K-MMSE consists of seven items that evaluate cognitive function across the dimensions of orientation, memory and registration, attention and calculation, recall, and language. The K-MMSE scores span from 0 to 30, with higher scores representing better cognitive function and lower scores indicating poorer cognitive performance. Continuous MMSE scores were dichotomised using a conventional cutoff of 24 points [30]. Decreased cognitive function was characterised by a K-MMSE score of 23 or lower. Normal cognitive function was characterised by a K-MMSE score of 24 or above.

### Other covariates

A structured questionnaire, facilitated by an interview, was administered to gather information on the participants’ sociodemographic (i.e. age, sex, and income) and lifestyle variables. According to the questionnaire responses, smoking status was categorised as ‘never-smokers’ for individuals who had never smoked cigarettes or smoked less than 100 cigarettes in their lifetime, ‘former-smokers’ for those who smoked 100 cigarettes or more but were not current smokers, and ‘current-smokers’ for individuals who had smoked 100 or more cigarettes and were currently smoking. Alcohol consumption status was classified as ‘never-drinker’ for individuals who had never consumed any alcoholic beverages in their lifetime, ‘former-drinker’ for those who had previously consumed alcoholic beverages but were not current consumers, and ‘current-drinker’ for those who had consumed alcoholic beverages more than once per month in the preceding year. Qualified personnel measured body weight and height during the physical examination, and the body mass index (BMI) was calculated as body weight in kilograms divided by height in meters squared. The consumption of energy, protein, fat, and carbohydrates was computed using a validated semi-quantitative food frequency questionnaire [31].

### Statistical analysis

The participants were classified into quartiles based on their PHAI scores, and all subsequent analyses were performed across these quartiles. Given that PHAI is an integer-based index, the resultant quartile distribution reflects a discrete scoring system and does not produce evenly proportioned groups.

The baseline characteristics of the study participants were presented as means ± standard deviations for continuous variables and numbers (percentages, %) for categorical variables. Population characteristics differences were tested using the chi-square test for categorical variables and Student’s *t*-test for continuous variables. Person-years of follow-up were computed from the date of initial assessment centre visit to the date of death or end of follow-up. The Cox proportional hazards model was employed to estimate the hazard ratios (HRs) and 95% confidence intervals (CIs) for mortality risk across PHAI quartiles. The crude model was modified. Model 1 was calibrated for age (continuous years) and sex. Model 2 was further adjusted for education level (elementary school or below, middle school, high school, college, or higher), household income (quartiles), smoking status (never smoker, former smoker, or current smoker), alcohol consumption status (never drinker, former drinker, or current drinker), body mass index (kg/m^2^, continuous), and total energy intake (kcal/day, continuous). Linear trends were tested by incorporating the median values of the quartiles of PHAI as a continuous variable in the model.

We conducted a sensitivity analysis to examine the robustness of our findings. We excluded hypotensive and hypoglycaemic individuals because PHAI’s cutoff does not account for hypotension and hypoglycaemia. All data analyses were conducted using SAS 9.4 (SAS Institute, Cary, NC, USA). The threshold for statistical significance was set at a *P* value of less than 0.05.

## Results

Out of 10,030 participants recruited to the KoGES, 6398 (63.8%) with complete data on PHAI and key covariates were included (Fig. 1) over an average follow-up period of 16.5 years (105,597.0 person-years). Table 1 shows the baseline characteristics of the study participants categorised by their PHAI scores in quartiles. Of the 6398 participants (mean age 51.23 years), 720 (11.25%) scored in the first quartile (Q1), 1191 (18.62%) in the second quartile (Q2), 1849 (28.90%) in the third quartile (Q3), and 2638 (41.23%) in the fourth quartile (Q4). Participants with higher PHAI scores were younger, more educated, possessed a higher household income, were non-smokers, and were current drinkers. In addition, higher PHAI scores were associated with a higher energy consumption, a relatively higher percentage of energy derived from proteins and fat, and a lower proportion of carbohydrates. The baseline characteristics of males and females are delineated in Supplementary Table 2, while the baseline characteristics of survival and death are outlined in Supplementary Table 3.

**Table 1:** Baseline characteristics of study participants according to the Physiology Healthy Aging Index

Characteristics	The Physiology Healthy Aging Index					*P* value^1)^
	Total	Q1 (lowest)	Q2	Q3	Q4 (highest)	
*N* (%)	6398 (100.00)^2)^	720 (11.25)	1191 (18.62)	1849 (28.90)	2638 (41.23)	
Age (years)	51.23 ± 0.1^2)^	58.75 ± 0.3	54.62 ± 0.3	51.54 ± 0.2	47.44 ± 0.1	< 0.001
	(40.0–69.0)	(40.0–69.0)	(40.0–69.0)	(40.0–69.0)	(40.0–69.0)	
Physiology Healthy Aging Index	8.96 ± 0.0	6.60 ± 0.0	8.00 ± 0.0	9.00 ± 0.0	10.00 ± 0.0	
	(3.0–10.0)	(3.0–7.0)	(8.0–8.0)	(9.0–9.0)	(10.0–10.0)	
Education level, *N* (%)						
Elementary school or below	1827 (28.56)	363 (50.42)	448 (37.62)	547 (29.58)	469 (17.78)	< 0.001
Middle school	1407 (21.99)	130 (18.06)	257 (21.58)	415 (22.44)	605 (22.93)	
High school	2162 (33.79)	151 (20.97)	318 (26.70)	579 (31.31)	1114 (42.23)	
College or above	1002 (15.66)	76 (10.56)	168 (14.11)	308 (16.66)	450 (17.06)	
Household income, *N* (%)						
Quartile 1	1881 (29.40)	347 (48.19)	441 (37.03)	597 (32.29)	460 (18.80)	< 0.001
Quartile 2	909 (14.21)	95 (13.19)	185 (15.53)	272 (14.71)	357 (13.53)	
Quartile 3	2258 (35.29)	182 (25.28)	362 (30.39)	601 (32.50)	1113 (42.19)	
Quartile 4	1350 (21.10)	96 (13.33)	203 (17.04)	379 (20.50)	672 (25.47)	
Smoking status, *N* (%)						
Never-smokers	3708 (57.96)	381 (52.92)	648 (54.41)	1020 (55.16)	1659 (62.89)	< 0.001
Former-smokers	1072 (16.76)	157 (21.81)	229 (19.23)	353 (19.09)	333 (12.62)	
Current-smokers	1618 (25.29)	182 (25.28)	314 (26.36)	476 (25.74)	646 (24.49)	
Drinking status, *N* (%)						
Never-drinker	2846 (44.48)	331 (45.97)	528 (44.33)	779 (42.13)	1208 (45.97)	0.0017
Former-drinker	384 (6.00)	62 (8.61)	68 (5.71)	112 (6.60)	132 (5.00)	
Current-drinker	3168 (49.52)	327 (45.42)	595 (49.96)	948 (51.27)	1298 (49.20)	
Body mass index (kg/m^2^)	24.61 ± 0.0	25.38 ± 0.1	25.03 ± 0.1	24.82 ± 0.1	24.07 ± 0.1	< 0.001
Waist circumference (cm)	82.10 ± 0.1	85.77 ± 0.3	84.22 ± 0.3	83.10 ± 0.2	79.44 ± 0.2	< 0.001
Hip circumference (cm)	94.09 ± 0.1	93.66 ± 0.3	94.21 ± 0.2	94.30 ± 0.1	94.01 ± 0.1	0.0616
SBP (mmHg)	120.20 ± 0.2	141.84 ± 0.7	134.27 ± 0.5	122.13 ± 0.3	106.58 ± 0.2	< 0.001
DBP (mmHg)	79.77 ± 0.1	89.56 ± 0.4	87.34 ± 0.3	81.74 ± 0.2	72.3 ± 0.2	< 0.001
Fasting blood glucose (mg/dL)	87.69 ± 0.3	109.81 ± 1.6	93.48 ± 0.8	84.48 ± 0.2	81.28 ± 0.1	< 0.001
Insulin (µIU/mL)	7.50 ± 0.1	8.25 ± 0.2	7.60 ± 0.1	7.64 ± 0.1	7.14 ± 0.1	< 0.001
Total cholesterol (mg/dL)	192.91 ± 0.5	200.32 ± 1.6	200.11 ± 1.1	193.15 ± 0.8	187.46 ± 0.6	< 0.001
Triglyceride (mg/dL)	160.59 ± 1.3	191.80 ± 4.9	177.82 ± 3.1	166.73 ± 2.4	140.70 ± 1.7	< 0.001
HDL-Cholesterol (mg/dL)	44.7 ± 0.1	44.23 ± 0.4	44.51 ± 0.3	44.22 ± 0.2	45.24 ± 0.2	0.0030
CRP (mg/dL)	0.22 ± 0.0	0.42 ± 0.0	0.27 ± 0.0	0.21 ± 0.0	0.16 ± 0.0	< 0.001
Creatinine (mg/dL)	0.85 ± 0.0	0.89 ± 0.0	0.87 ± 0.0	0.86 ± 0.0	0.83 ± 0.0	< 0.001
Fvc (L)	3.68 ± 0.0	3.06 ± 0.0	3.55 ± 0.0	3.74 ± 0.0	3.86 ± 0.0	< 0.001
Dietary intake						
Energy (kcal)	1994.47 ± 7.4	1939.63 ± 23.8	1985.39 ± 17.6	2000.81 ± 13.8	2009.10 ± 11.2	0.0418
Protein (g)	65.53 ± 0.3	63.28 ± 1.0	64.99 ± 0.8	65.17 ± 0.6	66.65 ± 0.5	0.0051
Fat (g)	31.04 ± 0.2	27.34 ± 0.6	29.65 ± 0.6	30.76 ± 0.4	32.87 ± 0.3	< 0.001
Carbohydrate (g)	357.67 ± 1.3	354.17 ± 4.3	358.72 ± 3.1	360.07 ± 2.4	356.47 ± 2.0	0.5227
Percentages of energy intake of^3)^						
Protein (%)	13.04 ± 0.0	12.91 ± 0.1	12.94 ± 0.1	12.92 ± 0.1	13.19 ± 0.0	< 0.001
Fat (%)	13.53 ± 0.1	12.19 ± 0.2	12.88 ± 0.2	13.33 ± 0.1	14.34 ± 0.1	< 0.001
Carbohydrate (%)	72.23 ± 0.1	73.57 ± 0.3	72.92 ± 0.2	72.52 ± 0.2	71.35 ± 0.1	< 0.001

*Q*, quartile; *Q1*, quartile 1 (lowest); *Q4*, quartile 4 (highest); *SBP*, systolic blood pressure; *DBP*, diastolic blood pressure; *HDL-Cholesterol*, high density lipoprotein cholesterol

^1)^*P* values were calculated using a chi-square test for categorical variables and linear regression analysis for continuous variables when comparing the baseline characteristics of the Physiology Healthy Aging Index

^2)^Data are presented as means ± standard deviation (SD) or number (%)

^3)^Percentages of energy intake from protein, fat, and carbohydrates were calculated as follows: protein intake and carbohydrate intake (g/day) × 4 kcal/total energy(kcal/day) × 100% and fat intake (g/day) × 9 kcal/total energy(kcal/day) × 100%

During the follow-up, 934 all-cause fatalities were recorded, including 778 age-related deaths, 353 attributed to cancer, and 184 resulting from CVD. Table 2 shows the HRs and 95% CIs for the correlation between PHAI and all-cause, age-related, cancer, and CVD mortalities. In fully adjusted models, HRs (95% CIs) for all-cause mortality in the upper categories were 0.82 (0.69–0.98), 0.50 (0.42–0.60), and 0.51 (0.41–0.63), respectively (*P* for trend < 0.001) compared to the lowest category. Comparable correlations were noted between age-related mortality, cancer mortality, and CVD mortality. In comparison to Q1, the HRs with 95% CIs for age-related mortality were 0.84 (0.70–1.02) for Q2, 0.51 (0.42–0.62) for Q3, and 0.51 (0.40–0.64) for Q4 (*P* for trend < 0.001). For cancer mortality, HRs were 0.78 (0.58–1.05) for Q2, 0.51 (0.37–0.69) for Q3, and 0.66 (0.48–0.92) for Q4 (*P* for trend = 0.0014). For CVD mortality, HRs were 0.92 (0.65–1.30) for Q2, 0.35 (0.23–0.52) for Q3, and 0.24 (0.13–0.44) for Q4 (*P* for trend < 0.001).

**Table 2:** Associations of the Physiology Healthy Aging Index with all-cause, age-related, cancer, and CVD mortality

HRs (95% CIs)	The Physiology Healthy Aging Index				*P* for trend
	Q1 (lowest)	Q2	Q3	Q4 (highest)	
No. of at risk	720	1,191	1,849	2,638	
Person-years	10,973	18,871	31,163	44,590	
Incidence rate per 1000 person-years	15.2	15.8	16.9	16.9	
All-cause					
No. of event	263	261	229	181	
Crude model	Reference	0.59 (0.50–0.71)	0.30 (0.25–0.36)	0.17 (0.14–0.21)	< 0.001
Model 1	Reference	0.81 (0.68–0.96)	0.51 (0.42–0.61)	0.52 (0.42–0.64)	< 0.001
Model 2	Reference	0.82 (0.69–0.98)	0.50 (0.42–0.60)	0.51 (0.41–0.63)	< 0.001
Age-related					
No. of event	220	222	192	144	
Crude model	Reference	0.60 (0.50–0.73)	0.30 (0.24–0.36)	0.16 (0.13–0.20)	< 0.001
Model 1	Reference	0.83 (0.68–1.00)	0.51 (0.42–0.62)	0.52 (0.41–0.65)	< 0.001
Model 2	Reference	0.84 (0.70–1.02)	0.51 (0.42–0.62)	0.51 (0.40–0.64)	< 0.001
Cancer					
No. of event	85	89	86	93	
Crude model	Reference	0.62 (0.46–0.83)	0.34 (0.25–0.46)	0.26 (0.19–0.34)	< 0.001
Model 1	Reference	0.77 (0.57–1.04)	0.53 (0.39–0.71)	0.69 (0.50–0.95)	0.0032
Model 2	Reference	0.78 (0.58–1.05)	0.51 (0.37–0.69)	0.66 (0.48–0.92)	0.0014
CVD					
No. of event	65	68	36	15	
Crude model	Reference	0.58 (0.41–0.82)	0.17 (0.11–0.26)	0.05 (0.03–0.09)	< 0.001
Model 1	Reference	0.91 (0.64–1.28)	0.34 (0.22–0.51)	0.23 (0.13–0.41)	< 0.001
Model 2	Reference	0.92 (0.65–1.30)	0.35 (0.23–0.52)	0.24 (0.13–0.44)	< 0.001

*Q*, quartile; *Q1*, quartile 1 (lowest); *Q4*, quartile 4 (highest); *CVD*, cardiovascular disease; *HR*, hazard ratio; *CI*, confidence interval

The *P* value for trend was calculated by including the median quintile values of the Physiology Healthy Aging Index as a continuous term in the model

^1)^Crude model was unadjusted; model 1 was adjusted for sex and age (years, continuous); model 2 was additionally adjusted Education level (Elementary school or below, Middle school, High school, College or above), Household income level (Quartiles), Smoking status (Never smoker, Former smoker, or Current smoker), Drinking status (Never-drinker, Former-drinker, or Current-drinker), body mass index (kg/m^2^, continuous), and total energy intake (kcal/day, continuous)

^2)^The *P* value for trend was calculated by including the median quintile values of the Physiology Healthy Aging Index as a continuous term in the model

To confirm the robustness and potential linearity of this association, we also performed a Cox regression treating the PHAI score as an ordinal variable (per 1-point increase). In the fully adjusted models, each 1-point increase in the PHAI score was significantly associated with a lower risk of all-cause mortality (HR 0.79, 95% CI = 0.75–0.83, *P* < 0.001), age-related mortality (HR 0.79, 95% CI = 0.74–0.84, *P* < 0.001), cancer mortality (HR 0.86, 95% CI = 0.79–0.95, *P* = 0.0017), and CVD mortality (HR 0.67, 95% CI = 0.60–0.75, *P* < 0.001). These results confirm a strong, dose-dependent relationship, supporting the findings from the primary quartile-based analysis.

The subgroup analyses revealed that the associations between PHAI and all-cause mortality, age-related mortality, and CVD mortality were similar for both men and women. Tables 3 and 4 present the sex-specific analyses. However, for cancer mortality, men in the higher categories exhibited a markedly reduced mortality risk compared to those in the lowest category. No apparent relationship was observed with cancer-related mortality in women.

**Table 3:** Associations of the Physiology Healthy Aging Index with all-cause, age-related, cancer, and CVD mortality in men

HRs (95% CIs)	Physiology Healthy Aging Index				P for trend
	Q1 (lowest)	Q2	Q3	Q4 (highest)	
No. of at risk	396	660	1000	1107	
Person-years	5932	10,212	16,778	18,489	
Incidence rate per 1000 person-years	15.0	15.5	16.8	16.7	
All-cause					
No. of event	156	163	137	108	
Crude model	Reference	0.63 (0.50–0.78)	0.30 (0.24–0.38)	0.22 (0.17–0.29)	< 0.001
Model 1	Reference	0.86 (0.69–1.07)	0.49 (0.39–0.62)	0.56 (0.43–0.73)	< 0.001
Model 2	Reference	0.87 (0.70–1.09)	0.48 (0.38–0.60)	0.52 (0.40–0.68)	< 0.001
Age-related					
No. of event	129	140	113	85	
Crude model	Reference	0.64 (0.50–0.81)	0.29 (0.23–0.37)	0.21 (0.16–0.27)	< 0.001
Model 1	Reference	0.87 (0.69–1.11)	0.49 (0.38–0.63)	0.55 (0.41–0.74)	< 0.001
Model 2	Reference	0.90 (0.71–1.15)	0.48 (0.37–0.63)	0.52 (0.39–0.71)	< 0.001
Cancer					
No. of event	57	63	57	50	
Crude model	Reference	0.62 (0.43–0.88)	0.32 (0.22–0.46)	0.26 (0.18–0.38)	< 0.001
Model 1	Reference	0.81 (0.56–1.16)	0.52 (0.36–0.75)	0.63 (0.42–0.95)	0.0030
Model 2	Reference	0.84 (0.58–1.21)	0.51 (0.35–0.74)	0.60 (0.40–0.91)	0.0013
CVD					
No. of event	32	34	20	9	
Crude model	Reference	0.58 (0.36–0.94)	0.17 (0.09–0.30)	0.08 (0.04–0.16)	< 0.001
Model 1	Reference	0.93 (0.57–1.51)	0.32 (0.18–0.58)	0.28 (0.13–0.60)	< 0.001
Model 2	Reference	0.95 (0.58–1.55)	0.33 (0.18–0.59)	0.29 (0.13–0.63)	< 0.001

*Q*, quartile; *Q1*, quartile 1 (lowest); *Q4*, quartile 4 (highest); *CVD*, cardiovascular disease; *HR*, hazard ratio; *CI*, confidence interval

^1)^Crude model was unadjusted; model 1 was adjusted for age (years, continuous); model 2 was additionally adjusted for education level (elementary school or below, middle school, high school, college or above), household income level (quartiles), smoking status (never smoker, former smoker, or current smoker), drinking status (never drinker, former drinker, or current drinker), body mass index (kg/m^2^, continuous), and total energy intake (kcal/day, continuous)

^2)^The *P* value for the trend was calculated by including the median quintile values of the Physiology Healthy Aging Index as a continuous term in the model

**Table 4:** Associations of the Physiology Healthy Aging Index with all-cause, age-related, cancer, and CVD mortality in women

HRs (95% CIs)	The Physiology Healthy Aging Index				P for trend
	Q1 (lowest)	Q2	Q3	Q4 (highest)	
No. of at risk	324	531	849	1531	
Person-years	5041	8659	14,385	26,101	
Incidence rate per 1000 person-years	15.6	16.3	16.9	17.0	
All-cause					
No. of event	107	98	92	73	
Crude model	Reference	0.55 (0.42–0.72)	0.30 (0.23–0.40)	0.13 (0.10–0.18)	< 0.001
Model 1	Reference	0.74 (0.56–0.98)	0.54 (0.41–0.72)	0.48 (0.34–0.67)	< 0.001
Model 2	Reference	0.76 (0.58–1.00)	0.54 (0.41–0.72)	0.49 (0.35–0.69)	< 0.001
Age-related					
No. of event	91	82	79	59	
Crude model	Reference	0.78 (0.58–1.06)	0.55 (0.40–0.75)	0.48 (0.33–0.70)	< 0.001
Model 1	Reference	0.76 (0.56–1.03)	0.55 (0.40–0.75)	0.47 (0.33–0.69)	< 0.001
Model 2	Reference	0.78 (0.58–1.06)	0.55 (0.40–0.75)	0.48 (0.33–0.70)	< 0.001
Cancer					
No. of event	28	26	29	43	
Crude model	Reference	0.60 (0.35–1.04)	0.37 (0.22–0.63)	0.30 (0.19–0.50)	< 0.001
Model 1	Reference	0.74 (0.43–1.27)	0.56 (0.33–0.96)	0.78 (0.45–1.35)	0.2925
Model 2	Reference	0.74 (0.43–1.28)	0.53 (0.31–0.91)	0.76 (0.43–1.34)	0.2298
CVD					
No. of event	33	34	16	6	
Crude model	Reference	0.57 (0.35–0.92)	0.16 (0.09–0.28)	0.03 (0.01–0.08)	< 0.001
Model 1	Reference	0.87 (0.54–1.42)	0.34 (0.18–0.62)	0.18 (0.07–0.45)	< 0.001
Model 2	Reference	0.87 (0.53–1.41)	0.34 (0.19–0.64)	0.18 (0.07–0.46)	< 0.001

*Q*, quartile; *Q1*, quartile 1 (lowest); *Q4*, quartile 4 (highest); *CVD*, cardiovascular disease; *HR*, hazard ratio; *CI*, confidence interval

^1)^Crude model was unadjusted; model 1 was adjusted for age (years, continuous); model 2 was additionally adjusted for education level (elementary school or below, middle school, high school, college or above), household income level (quartiles), smoking status (never smoker, former smoker, or current smoker), drinking status (never drinker, former drinker, or current drinker), body mass index (kg/m^2^, continuous), and total energy intake (kcal/day, continuous)

^2)^The *P* value for the trend was calculated by including the median quintile values of the Physiology Healthy Aging Index as a continuous term in the model

A notable strength of our study is the acquisition of both the baseline PHAI and the PHAI at the final follow-up. Consequently, we calculated the change in the PHAI score by subtracting the baseline score from the score recorded at the last follow-up over an approximate period of 20 years. The alteration in PHAI scores was divided into tertiles: ‘Accelerated agers’ for individuals whose scores decreased from baseline to follow-up, ‘Resilient agers’ for those whose scores increased, and ‘Stable agers’ for those whose scores remained constant. Associations with all-cause mortality, age-related mortality, cancer mortality, and CVD mortality were analysed (Table 5). In the fully adjusted analysis, relative to the decreased group, the increased and stable groups had significantly lower risks of all-cause, age-related, cancer, and CVD mortality, with the decrease in risk being more pronounced in the increased group.

**Table 5:** Associations of changes in the Physiology Healthy Aging Index with all-cause, age-related, cancer, and CVD mortality

HRs (95% CIs)	Changes in the Physiology Healthy Aging Index			*P* for trend
	Accelerated agers	Stable agers	Resilient agers	
No. of at risk	1627	3681	1090	
Person-years	25,797	60,262	19,538	
Incidence rate per 1000 person-years	15.9	16.4	17.9	
All-cause				
No. of event	227	601	106	
Crude model	Reference	1.11 (0.95–1.29)	0.61 (0.48–0.77)	0.0006
Model1	Reference	0.99 (0.85–1.15)	0.49 (0.39–0.62)	< 0.001
Model2	Reference	1.03 (0.88–1.20)	0.51 (0.40–0.64)	< 0.001
Model3	Reference	0.65 (0.54–0.77)	0.21 (0.16–0.28)	< 0.001
Age-related				
No. of event	186	501	91	
Crude model	Reference	1.10 (0.93–1.31)	0.62 (0.48–0.79)	0.0016
Model1	Reference	0.98 (0.82–1.16)	0.49 (0.38–0.63)	< 0.001
Model2	Reference	1.01 (0.85–1.20)	0.50 (0.39–0.65)	< 0.001
Model3	Reference	0.61 (0.50–0.74)	0.19 (0.14–0.27)	< 0.001
Cancer				
No. of event	102	228	23	
Crude model	Reference	0.97 (0.77–1.22)	0.30 (0.19–0.47)	< 0.001
Model1	Reference	0.90 (0.71–1.14)	0.25 (0.16–0.39)	< 0.001
Model2	Reference	0.93 (0.73–1.18)	0.26 (0.16–0.41)	< 0.001
Model3	Reference	0.63 (0.48–0.83)	0.12 (0.07–0.21)	< 0.001
CVD				
No. of event	42	109	33	
Crude model	Reference	1.07 (0.75–1.53)	0.99 (0.63–1.56)	0.9884
Model1	Reference	0.85 (0.59–1.22)	0.69 (0.44–1.10)	0.1162
Model2	Reference	0.83 (0.58–1.20)	0.64 (0.40–1.02)	0.0610
Model3	Reference	0.44 (0.29–0.66)	0.19 (0.10–0.35)	< 0.001

*CVD*, cardiovascular disease; *HR*, hazard ratio; *CI*, confidence interval

^1)^Crude model was unadjusted; model 1 was adjusted for sex and age (years, continuous); model 2 was additionally adjusted Education level (Elementary school or below, Middle school, High school, College or above), Household income level (Quartiles), Smoking status (Never smoker, Former smoker, or Current smoker), Drinking status (Never-drinker, Former-drinker, or Current-drinker), Menopause status (Yes or No), body mass index (kg/m^2^, continuous), and total energy intake (kcal/day, continuous); model 3 was additionally adjusted baseline survey the Physiology Healthy Aging Index (continuous)

^2)^The *P* value for trend was calculated by including the median quintile values of the Physiology Healthy Aging Index as a continuous term in the model

**Fig. 2:**
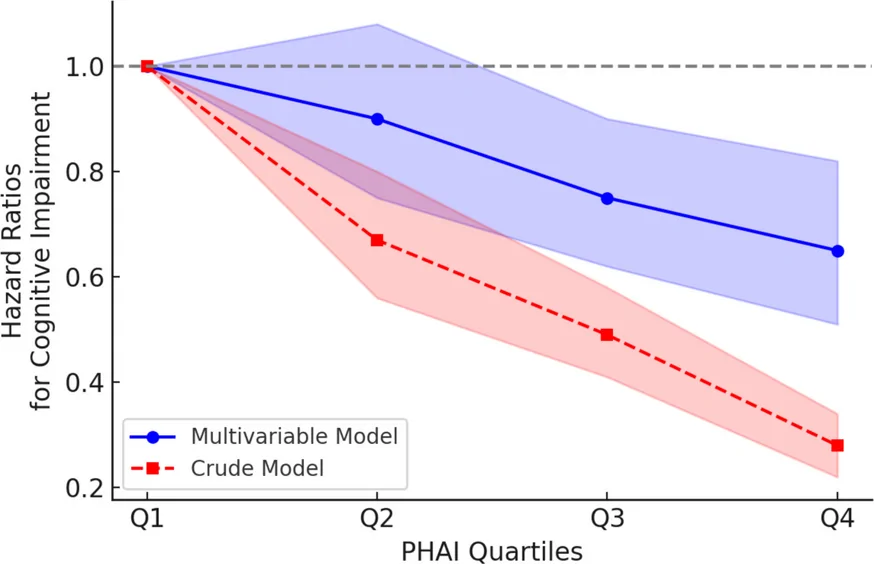
Hazard ratios with 95% confidence intervals (CI) of cognitive impairment incidence by categories of the Physiology Healthy Aging Index. (Crude model, red; Multivariable adjusted model, blue) Multivariable adjusted was adjusted for age (years, continuous), sex, education level (elementary school or below, middle school, high school, college or above), household income level (quartiles), smoking status (never smoker, former smoker, or current smoker), drinking status (never-drinker, former-drinker, or current-drinker), body mass index (kg/m^2^, continuous), and total energy intake (kcal/day, continuous).

Given that the PHAI comprises elements associated with physiological aging, we aimed to investigate its correlation with cognitive function, which is independently linked to aging. Therefore, we calculated the PHAI scores for 5011 participants who commenced cognitive function testing in wave 4, excluding individuals with missing PHAI components or covariates, abnormal energy intake, incomplete follow-up, or abnormal cognitive function (Supplementary Fig. 1). Figure 2 illustrates a significant correlation between the PHAI and the occurrence of cognitive impairment (for both models, *P* for trend < 0.0001). Subsequent modification resulted in a slight reduction of the association’s magnitude within the multivariate model. Specific data are shown in Supplementary Table 4.

In the sensitivity analyses, we excluded participants with hypotension and hypoglycaemia, as these conditions may result in elevated PHAI scores. The exclusion did not significantly alter the results for all-cause, age-related, cancer, or CVD mortality (refer to Supplementary Fig. 2 for the participant flow diagram and Supplementary Table 5 for the results).

## Discussion

In this extensive prospective cohort study of older Korean adults, we developed a PHAI based on five clinically measurable biomarkers: systolic blood pressure, fasting blood glucose, creatinine, forced vital capacity, and C-reactive protein levels. Our findings demonstrated that higher PHAI scores, reflecting healthier physiological aging, were significantly correlated with a reduced risk of all-cause, age-related cancer, and CVD mortality. Moreover, the persistence of PHAI over approximately 20 years has been associated with a decreased mortality risk, highlighting its potential as a dynamic aging indicator.

The biological rationale for these associations is multifaceted. Systolic blood pressure indicates cardiovascular health and arterial rigidity, which are essential components in vascular aging [32]. Fasting blood glucose levels indicate metabolic function and insulin sensitivity, which are crucial for healthy aging trajectories [33]. Serum creatinine functions as a marker of kidney function, which declines with age and significantly affects overall health outcomes [34]. Forced vital capacity represents respiratory function and has been consistently linked to longevity across diverse populations [35, 36]. C-reactive protein, an inflammatory marker, captures the chronic low-grade inflammation characteristic of aging, often termed ‘inflammaging’ [20]. The integration of these biomarkers into a single index provides a comprehensive assessment of physiological aging, transcending the limitations of individual markers.

We acknowledge the intriguing link between PHAI and cognitive impairment, which suggests systemic aging processes. Several potential pathways likely mediate this connection. First, vascular mechanisms are critical; components like elevated systolic blood pressure (in PHAI) directly impair cerebral perfusion and blood–brain barrier integrity. Second, metabolic pathways play a key role, as elevated fasting glucose (in PHAI) promotes neuronal dysfunction via insulin resistance and advanced glycation end-products. Finally, these pathways are interconnected by systemic inflammation (measured by CRP in PHAI), which fosters a neuroinflammatory environment detrimental to cognitive health. Thus, PHAI likely captures the cumulative burden of these shared systemic processes that simultaneously impact peripheral physiology and brain aging [37–39].

The PHAI distinguishes itself from existing aging indices in several scientifically meaningful ways beyond accessibility. First, while the original HAI includes neurocognitive function, our substitution of C-reactive protein is not merely pragmatic but captures ‘inflammaging’—a fundamental aging hallmark increasingly recognised as a driver of multi-organ decline [20]. This provides complementary rather than redundant information: whereas HAI directly measures one aging outcome (cognitive function), PHAI measures an upstream mechanistic process (chronic inflammation) that contributes to multiple aging phenotypes. Our finding that PHAI predicts cognitive impairment (Fig. 2) despite not including cognitive measures validates this approach and suggests shared biological pathways. Second, we introduce a novel trajectory classification framework (Accelerated/Stable/Resilient agers) with striking prognostic discrimination. Resilient agers showed a 79% lower mortality risk compared to accelerated agers (HR 0.21, 95% CI 0.16–0.28)—a magnitude of effect exceeding that reported for single-point HAI measurements [40]. This categorical approach has immediate clinical translation potential: clinicians can readily identify and monitor patients transitioning between aging trajectories, enabling timely intervention before irreversible decline occurs. Third, compared to Zhu et al.’s 17-biomarker PAI derived through complex machine learning [41], our 5-component PHAI achieves comparable predictive performance with dramatically enhanced interpretability and implementability. The trade-off between model complexity and practical utility favours simpler indices in population health applications, particularly in resource-limited settings where comprehensive biomarker panels are infeasible. Fourth, our 20-year follow-up in an Asian cohort addresses a critical evidence gap. While HAI validation has focused primarily on Western populations [14–18], differences in aging trajectories, disease patterns, and biomarker distributions across ethnicities necessitate population-specific validation. Our findings demonstrate that physiological aging indices maintain predictive validity in East Asian populations, supporting their potential as universal aging assessment tools.

The PHAI distinguishes itself from current aging indices in significant respects. In contrast to complex biological age calculators necessitating specialised laboratory tests or the Frailty Index, which depends on deficit accumulation across various domains [42, 43], the PHAI utilises merely five routinely assessed clinical parameters. This simplicity enhances their practical applicability in routine clinical care and population health monitoring. Various biological aging measures have been developed, encompassing the epigenetic clock, telomere length assessment, and composite biomarker panels [44, 45]. Although these approaches offer valuable insights into the aging mechanisms, they frequently require advanced laboratory techniques and incur substantial expenses. In contrast, the PHAI leverages standard clinical measurements commonly found in most healthcare settings, rendering it appropriate for implementation in resource-constrained environments or extensive screening programs.

This study’s longitudinal nature provides distinct insights into aging trajectories. Although cross-sectional studies have shown a correlation between individual biomarkers and health outcomes, our 20-year follow-up study demonstrated that alterations in physiological aging patterns, as captured by PHAI trajectories, provided more insightful information than single-point assessments. This finding supports the notion that aging is a dynamic, alterable process rather than a fixed trajectory [46].

The clinical utility of PHAI surpasses mere mortality prediction. Its simplicity and reliance on standard clinical measurements render it an optimal instrument for identifying individuals at high risk of adverse outcomes who may benefit from targeted interventions. Clinicians may utilise declining PHAI scores as precursory indicators, necessitating enhanced surveillance or preventive measures prior to the manifestation of an overt disease. The finding that PHAI improvement or maintenance over time correlates with a reduced mortality risk carries substantial implications for healthy aging interventions, suggesting that lifestyle changes or medical interventions targeting component biomarkers could alter aging trajectories [47].

From a public health perspective, the PHAI offers numerous benefits for population-level health monitoring. Its straightforward calculation allows easy implementation in electronic health records and health surveillance systems. Policymakers can utilise population-level PHAI distributions to assess the overall health status of aging communities and evaluate the effectiveness of health promotion programs. Furthermore, this index can guide resource allocation by pinpointing geographical regions or demographic groups with disproportionately adverse physiological aging profiles.

Recent validation studies of HAI in European populations, including the 15-year Doetinchem Cohort Study, have substantiated its predictive capacity for mortality, morbidity, and disability [16], while multi-ethnic cohort studies from Singapore have demonstrated the association of HAI with various health-related outcomes beyond mortality [48]. The findings from diverse populations support the robustness of the composite physiological aging indices and validate our approach for developing a culturally adapted index for Korean older adults.

Numerous biological aging measures have been developed, such as the epigenetic clock, telomere length assessment, and composite biomarker panels [44, 45]. While these methodologies provide significant insights into the aging mechanisms, they frequently necessitate advanced laboratory techniques and incur substantial expenses. In contrast, the PHAI utilises standard clinical measurements accessible in most healthcare settings, rendering it especially appropriates for implementation in resource-limited environments or extensive screening initiatives.

This study possesses various methodological advantages. The large sample size and prospective design with extended follow-up provided robust evidence for the observed associations. However, it is critical to emphasise the observational nature of our design. While these findings demonstrate a strong correlation, they do not establish causation, and we cannot exclude the possibility of residual confounding. Comprehensive mortality data, including cause-specific analyses, enhances the clinical significance of the findings. Additionally, the ability to monitor PHAI variations over time yields valuable insights into the aging dynamics that cross-sectional studies cannot offer, though these temporal relationships must also be interpreted as associations rather than definitive causal pathways. However, this study has several limitations that require attention. As an observational study, we were unable to establish causal relationships between PHAI and mortality outcomes, despite the temporal sequence and biological plausibility of the associations. Despite adjusting for numerous potential confounders, residual confounding from unmeasured variables may still exist. The quartile classification did not yield perfectly balanced groups because the PHAI is an integer-based index; this should be considered when interpreting comparisons across quartiles. We chose quartiles for our primary analysis for several reasons. First, the PHAI score distribution was discrete (integer scores from 3 to 10) and highly skewed, with a large proportion of the cohort (41.23%) achieving the maximum score of 10. Treating this non-uniform, discrete data as truly continuous would not accurately represent the data structure. Second, from a clinical perspective, quartiles provide actionable, distinct risk categories for patient stratification and communication, which is often more interpretable than a per-unit increase in risk. Third, this categorical approach aligns with the precedent set by prior aging indices, including the original HAI, facilitating direct comparison with existing literature. Finally, our findings are derived from a cohort of older Korean adults, which critically limits the generalisability of the PHAI to other ethnic groups or younger demographics. This limitation is not trivial, given that substantial evidence demonstrates significant ethnic and racial variations in the distributions and predictive values of the biomarkers comprising the PHAI (e.g. CRP, creatinine) [49, 50]. Therefore, the specific weightings and thresholds established in our index may not be directly applicable to non-Korean or non-Asian populations, warranting further validation and recalibration in diverse cohorts.

The PHAI, although extensive in its scope, captures only a fraction of age-related physiological changes. Significant domains such as physical performance and sensory impairment are not explicitly reflected in the index [51]. The equal weighting of the five components, while statistically derived, may not accurately reflect the relative significance of each biomarker among individuals or populations. The possibility of survivor bias cannot be entirely excluded, as individuals who participated in the study for the entire 20-year follow-up may represent a healthier subset of the original cohort.

Multiple research avenues emerged from our findings. External validation of the PHAI in diverse populations, including different ethnicities and age demographics, is crucial for confirming its broader applicability. Cross-cultural studies may clarify whether the observed biomarker relationships are universal or population-specific, potentially resulting in culturally adapted versions of the index.

Methodological improvements could enhance the predictive accuracy of PHAIs. Machine learning approaches may optimise biomarker weighting or identify nonlinear relationships among components [52]. The inclusion of supplementary biomarkers such as albumin, haemoglobin, or lipid profiles could improve the index’s comprehensiveness. Examining sex-specific or age-stratified versions may uncover differential aging patterns among demographic groups.

Longitudinal studies examining PHAI trajectories concerning specific health outcomes, healthcare utilisation, and quality of life metrics would yield enhanced understanding of the index’s clinical utility. Research on the responsiveness of PHAI to various interventions, such as lifestyle modifications, pharmacological treatments, and preventive care programs, would validate its significance as an outcome measure in clinical trials and public health interventions.

## Conclusions

In this extensive prospective cohort study of older Korean adults, we developed and validated the PHAI based on clinically relevant biomarkers. Our findings indicated that higher PHAI scores were significantly associated with a reduced risk of all-cause and cause-specific mortality, encompassing age-related, cancer-related, and cardiovascular deaths. Furthermore, longitudinal changes in the PHAI scores predicted mortality risk, underscoring the significance of maintaining or improving physiological health during aging.

Additionally, the observed association between the PHAI and cognitive function suggests that this index may function as a holistic indicator of overall aging status.

While the PHAI demonstrates potential as a comprehensive aging assessment tool, further research is necessary to enhance its components, validate its efficacy across diverse populations, and determine its effectiveness in guiding clinical decision-making and public health interventions. As our comprehension of aging mechanisms advances, the PHAI provides a foundation for developing more sophisticated and personalised strategies for healthy aging assessment and intervention.

As global healthcare systems face the challenges of population aging, simple yet robust tools, such as the PHAI, may be essential in promoting successful aging and alleviating the impact of age-related diseases. The ultimate goal is to convert scientific insights into practical tools that enhance the health and quality of life for aging populations.

## Supplementary Information

Below is the link to the electronic supplementary material.

Supplementary file1 (DOCX 98 kb)

## References

[1] United Nations, Department of Economic and Social Affairs, Population Division. World Population Prospects 2022: summary of results. New York: United Nations; 2022.

[2] Richter F. Which countries have the oldest populations? World Economic Forum 2023. https://www.weforum.org/stories/2023/02/world-oldest-populations-asia-health. Accessed 28 Aug 2025.

[3] United Nations, Department of Economic and Social Affairs. World Social Report 2023: leaving no one behind in an aging world. New York: United Nations; 2023.

[4] Statistics Korea. Future population projections for Korea: 2022–2070. Daejeon: Statistics Korea; 2021

[5] Gianfredi V, Nucci D, Pennisi F, Maggi S, Veronese N, Soysal P. Aging, longevity, and healthy aging: the public health approach. Aging Clin Exp Res. 2025;37:1–12. 10.1007/s40520-025-03021-8 PMC12006278 40244306

[6] Beard JR, Officer A, De Carvalho IA, Sadana R, Pot AM, Michel JP, et al. The World report on ageing and health: a policy framework for healthy ageing. Lancet. 2016;387(10033):2145–54. 10.1016/S0140-6736(15)00516-4 PMC4848186 26520231

[7] Lafortune L, Martin S, Kelly S, et al. Behavioural risk factors in mid-life associated with successful aging, disability, dementia and frailty in later life: a rapid systematic review. PLoS One. 2016;11:e0144405. 10.1371/journal.pone.0144405 PMC4742275 26845035

[8] Mathur A, Taurin S, Alshammary S. New insights into methods to measure biological age: a literature review. Front Aging. 2024;5:1395649. 10.3389/fragi.2024.1395649 PMC11688636 39743988

[9] Li Z, Zhang W, Duan Y, et al. Progress in biological age research. Front Public Health. 2023;11:1074274. 10.3389/fpubh.2023.1074274 PMC10130645 37124811

[10] Bernabeu E, McCartney DL, Gadd DA. Refining epigenetic prediction of chronological and biological age. Genome Med. 2023;15:12. 10.1186/s13073-023-01161-y PMC9976489 36855161

[11] Damluji AA, Nanna MG, Rymer J, Kochar A, Lowenstern A, Baron SJ, et al. Chronological vs biological age in interventional cardiology: a comprehensive approach to care for older adults: JACC family series. Cardiovascul Intervent. 2024;17(8):961–78. 10.1016/j.jcin.2024.01.284 PMC11097960 38597844

[12] Tao X, Zhu Z, Wang L, Li C, Sun L, Wang W, et al. Biomarkers of aging and relevant evaluation techniques: a comprehensive review. Aging Dis. 2024;15:977. 10.14336/AD.2023.00808-1 PMC11081160 37611906

[13] LeBrasseur NK. Hungry for biomarkers of aging. Aging Cell. 2024;23:e14158. 10.1111/acel.14158 PMC11019142 38532727

[14] Newman AB, Boudreau RM, Naydeck BL, Fried LF, Harris TB. A physiologic index of comorbidity: relationship to mortality and disability. J Gerontol A Biol Sci Med Sci. 2008;63:603–9. 10.1093/gerona/63.6.603 PMC2496995 18559635

[15] Zhang H, Zhu Y, Hao M, et al. The modified healthy aging index is associated with mortality and disability: the Rugao Longevity and Aging Study. Gerontol. 2021;67:572–80. 10.1159/000513931 34000721

[16] Dieteren CM, Samson LD, Schipper M, et al. The healthy aging index analysed over 15 years in the general population: the Doetinchem cohort study. Prev Med. 2020;39:106193. 10.1016/j.ypmed.2020.106193 32653354

[17] McCabe EL, Larson MG, Lunetta KL, Newman AB, Cheng S, Murabito JM. Association of an index of healthy aging with incident cardiovascular disease and mortality in a community-based sample of older adults. J Gerontol A Biol Sci Med Sci. 2016;71:1695–701. 10.1093/gerona/glw077 PMC5106860 27117172

[18] Zhuang Z, Zhao Y, Huang N, et al. Associations of healthy aging index and all-cause and cause-specific mortality: a prospective cohort study of UK Biobank participants. GeroScience. 2024;46:1241–57. 10.1007/s11357-023-00891-6 PMC10828282 37526907

[19] Sanders JL, Minster RL, Barmada MM, et al. Heritability of and mortality prediction with a longevity phenotype: the healthy aging index. J Gerontol A Biol Sci Med Sci. 2014;69:479–85. 10.1093/gerona/glt117 PMC3968826 23913930

[20] Franceschi C, Bonafè M, Valensin S, et al. <article-title update="added">Inflamm‐aging: an evolutionary perspective on immunosenescence. Ann N Y Acad Sci. 2000;908:244–54. 10.1111/j.1749-6632.2000.tb06651.x 10911963

[21] Kim Y, Han B-G, Group K. Cohort profile: the Korean Genome and Epidemiology Study (KoGES) consortium. Int J Epidemiol. 2017;46:e20–e20. 10.1093/ije/dyv316 PMC5837648 27085081

[22] Kim H-L, Lee EM, Ahn SY, et al. The 2022 focused update of the 2018 Korean Hypertension Society Guidelines for the management of hypertension. Clin Hypertens. 2023;29:11. 10.1186/s40885-023-00234-9 PMC9930285 36788612

[23] Moon JS, Kang S, Choi JH, et al. 2023 clinical practice guidelines for diabetes management in Korea: full version recommendation of the Korean diabetes association. Diabetes Metab J. 2024;48:546–708. 10.4093/dmj.2024.0249 PMC11307112 39091005

[24] Locatelli F, Nissenson AR, Barrett BJ, et al. Clinical practice guidelines for anemia in chronic kidney disease: problems and solutions. A position statement from Kidney Disease: Improving Global Outcomes (KDIGO). Kidney Int. 2008;74:1237–40. 10.1038/ki.2008.299 18596731

[25] Pearson TA, Mensah GA, Alexander RW, Anderson JL, Cannon III RO, Criqui M, Fadl YY, Fortmann SP, Hong Y, Myers GL, Rifai N. Markers of inflammation and cardiovascular disease: application to clinical and public health practice: a statement for healthcare professionals from the Centers for Disease Control and Prevention and the American Heart Association. circulation. 2003 Jan 28;107(3):499-511. 10.1161/01.cir.0000052939.59093.45 12551878

[26] Jung EJ, Lee SH, Na JC, Kim SS. Introduction to data on death causes linked with the Korean Genome and Epidemiology Study (KoGES) epidemiological data. Public Health Wkly Rep. 2018;11:159–62.

[27] Statistics Korea: Korean standard classification of diseases and causes of death, 6th revision edn. Daejeon: Statistics Korea; 2010.

[28] World Health Organization. International statistical classification of diseases and related health problems, 10th revision, 5th edition, 2016 edition. Geneva: World Health Organization; 2015.

[29] Kang Y, NA DL, Hahn S. A validity study on the Korean Mini-Mental State Examination (K-MMSE) in dementia patients. Journal of the Korean neurological association. 1997:300-8.

[30] Kukull W, Larson E, Teri L, Bowen J, McCormick W, Pfanschmidt M. The mini-mental state examination score and the clinical diagnosis of dementia. J Clin Epidemiol. 1994;47:1061–7. 10.1016/0895-4356(94)90122-8 7730909

[31] Park S-J, Lyu J, Lee K, Lee H-J, Park H-Y. Nutrition survey methods and food composition database update of the Korean Genome and Epidemiology Study. Epidemiol Health. 2024;46:e2024042. 10.4178/epih.e2024042 PMC11417449 38574826

[32] Mitchell GF. Arterial stiffness and hypertension. Hypertension. 2014;64:13–8. 10.1161/HYPERTENSIONAHA.114.00921 PMC4063409 24752432

[33] Barzilai N, Crandall JP, Kritchevsky SB, Espeland MA. Metformin as a tool to target aging. Cell Metab. 2016;23:1060–5. 10.1016/j.cmet.2016.05.011 PMC5943638 27304507

[34] Coresh J, Selvin E, Stevens LA, et al. Prevalence of chronic kidney disease in the United States. JAMA. 2007;298:2038–47. 10.1001/jama.298.17.2038 17986697

[35] Hj S. Pulmonary function is a long-term predictor of mortality in the general population: 29-year follow-up of the Buffalo Health Study. Chest. 2000;118:656–64. 10.1378/chest.118.3.656 10988186

[36] Sin DD, Wu L, Man SP. The relationship between reduced lung function and cardiovascular mortality: a population-based study and a systematic review of the literature. Chest. 2005;127:1952–9. 10.1378/chest.127.6.1952 15947307

[37] Iadecola C. The pathobiology of vascular dementia. Neuron. 2013;80:844–66. 10.1016/j.neuron.2013.10.008 PMC3842016 24267647

[38] Cai W, Ramdas M, Zhu L, Chen X, Striker GE, Vlassara H. Oral advanced glycation endproducts (AGEs) promote insulin resistance and diabetes by depleting the antioxidant defenses AGE receptor-1 and sirtuin 1. Proc Natl Acad Sci U S A. 2012;109:15888–93. 10.1073/pnas.1205847109 PMC3465382 22908267

[39] Walker KA, Gottesman RF, Wu A, et al. Systemic inflammation during midlife and cognitive change over 20 years: the ARIC study. Neurology. 2019;92:e1256–67. 10.1212/WNL.0000000000007094 PMC6511107 30760633

[40] O’Connell MD, Marron MM, Boudreau RM, et al. Mortality in relation to changes in a healthy aging index: the health, aging, and body composition study. J Gerontol A Biol Sci Med Sci. 2019;74:726–32. 10.1093/gerona/gly114 PMC6477637 29733331

[41] Zhu Z, Lyu J, Hao X, et al. Estimation of physiological aging based on routine clinical biomarkers: a prospective cohort study in elderly Chinese and the UK Biobank. BMC Med. 2024;22:552. 10.1186/s12916-024-03769-2 PMC11583456 39578829

[42] Rockwood K, Mitnitski A. Frailty in relation to the accumulation of deficits. J Gerontol A Biol Sci Med Sci. 2007;62:722–7. 10.1093/gerona/62.7.722 17634318

[43] Fried LP, Tangen CM, Walston J, et al. Frailty in older adults: evidence for a phenotype. J Gerontol A Biol Sci Med Sci. 2001;56:M146–57. 10.1093/gerona/56.3.m146 11253156

[44] Horvath S. DNA methylation age of human tissues and cell types. Genome Biol. 2013;14:3156. 10.1186/gb-2013-14-10-r115 PMC4015143 24138928

[45] Blackburn EH, Epel ES, Lin J. Human telomere biology: a contributory and interactive factor in aging, disease risks, and protection. Science. 2015;350:1193–8. 10.1126/science.aab3389 26785477

[46] Kuh D. A life course approach to healthy aging, frailty, and capability. J Gerontol A Biol Sci Med Sci. 2007;62:717–21. 10.1093/gerona/62.7.717 17634317

[47] Seals DR, Justice JN, LaRocca TJ. Physiological geroscience: targeting function to increase health span and achieve optimal longevity. J Physiol. 2016;594:2001–24. 10.1113/jphysiol.2014.282665 PMC4933122 25639909

[48] Fauzi NBM, Huang X, Cheng LJ, Luo N, Hilal S. Association of a healthy aging index with health-related outcomes in a multi-ethnic cohort from Singapore. BMC Geriatr. 2024;24:508. 10.1186/s12877-024-05099-7 PMC11165847 38862903

[49] Albert MA, Glynn RJ, Buring J, Ridker PM. C-reactive protein levels among women of various ethnic groups living in the United States (from the Women’s Health Study). Am J Cardiol. 2004;93(10):1238–42. 10.1016/j.amjcard.2004.01.067 15135696

[50] Flamant M, Vidal-Petiot E, Metzger M, et al. Performance of GFR estimating equations in African Europeans: basis for a lower race-ethnicity factor than in African Americans. Am J Kidney Dis. 2013;62:182–4. 10.1053/j.ajkd.2013.03.015 23608543

[51] Blodgett J, Theou O, Kirkland S, Andreou P, Rockwood K. The association between sedentary behaviour, moderate–vigorous physical activity and frailty in NHANES cohorts. Maturitas. 2015;80:187–91. 10.1016/j.maturitas.2014.11.010 25542406

[52] Putin E, Mamoshina P, Aliper A, et al. Deep biomarkers of human aging: application of deep neural networks to biomarker development. Aging. 2016;8:1021. 10.18632/aging.100968 PMC4931851 27191382

