# Supplementary material for: A novel clinical biomarker-based Physiology Healthy Aging Index and risk of all-cause and cause-specific mortality: A 20-year prospective cohort study
Source: GeroScience. 2025 Dec 6;48(5):6989–7005. doi: 10.1007/s11357-025-02025-6 (PMC13601385; doi:10.1007/s11357-025-02025-6)
Supplement: Supplementary file 1 [file 11357_2025_2025_MOESM1_ESM.docx]

**Supplementary Content**

**Supplementary Table 1.** Cutoff points were used to categorise each indicator of the Physiological Healthy Aging Index in the KoGES (Ansan-Ansung) study.

**Supplementary Table 2.** Baseline characteristics of the participants in the KoGES (Ansan-Ansung) Study.

**Supplementary Table 3.** Baseline characteristics according to survivors and deaths of participants in the Ansan (Ansan-Ansung) study.

**Supplementary Figure 1.** Flow diagram of the study participants with cognitive impairment.

**Supplementary Table 4.** Hazard ratios with 95% confidence intervals (CI) of cognitive impairment incidence by Physiology Healthy Aging Index category

**Supplementary Figure 2.** Sensitivity analysis study participants: Flow diagram.

**Supplementary Table 5.** Associations between the physiologically healthy aging index and all-cause, age-related, cancer, and CVD mortality in a sensitivity analysis of study participants

**Supplementary Table 1.** Cutoff points were used to categorise each indicator of the Physiological Healthy Aging Index in the KoGES (Ansan-Ansung) study

| Score for group | SBP, mmHG | FBS, mg/dL | Creatinine, mg/dL | Fvc, L | | CRP  mg/L | hsCRP  mg/L |
| --- | --- | --- | --- | --- | --- | --- | --- |
|  |  |  |  | Men | Women |  |  |
| 0= lest healthy | ≥ 140 | ≥ 126 | > 2.0 | < 3.2 | < 2.1 | >1 | >3 |
| 1=intermediate | 120-139 | 100-125 | 1.4-2.0 | 3.2-3.8 | 2.1-2.6 | 0.5-1 | 1-3 |
| 2=healthiest | < 120 | < 100 | < 1.4 | > 3.8 | > 2.6 | < 0.5 | < 1 |

**Supplementary Table 2.** Baseline characteristics of the participants in the KoGES (Ansan-Ansung) Study.

| Characteristics | Total | Men | Women | P-value^1)^ |
| --- | --- | --- | --- | --- |
| N (%) | 6,398 (100.00)^2)^ | 3,163 (49.44) | 3,235 (50.56) |  |
| Age (years) | 51.23 ± 0.1^2)^ | 50.63 ± 0.2 | 51.82 ± 0.2 | <0.001 |
|  | (40.0-69.0) | (40.0-69.0) | (40.0-69.0) |  |
| Physiology Healthy Aging Index | 8.96 ± 0.0 | 8.84 ± 0.0 | 9.07 ± 0.0 | <0.001 |
|  | (3.0-10.0) | (3.0-10.0) | (3.0-10.0) |  |
| Education level, N (%) |  |  |  |  |
| Elementary school or below | 1,827 (28.56) | 519 (16.41) | 1,308 (40.43) | <0.001 |
| Middle school | 1,407 (21.99) | 662 (20.93) | 745 (23.03) |  |
| High school | 2,162 (33.79) | 1,216 (38.44) | 946 (29.24) |  |
| College or above | 1,002 (15.66) | 766 (24.22) | 236 (7.30) |  |
| Household income, N (%) ^3)^ |  |  |  |  |
| Quartile 1 | 1,881 (29.40) | 704 (22.26) | 1,177 (36.38) | <0.001 |
| Quartile 2 | 909 (14.21) | 418 (13.22) | 491 (15.18) |  |
| Quartile 3 | 2,258 (35.29) | 1,208 (38.19) | 1,050 (32.46) |  |
| Quartile 4 | 1,350 (21.10) | 833 (26.34) | 517 (15.98) |  |
| Smoking status, N (%) |  |  |  |  |
| Never- smokers | 3,708 (57.96) | 629 (19.89) | 3,079 (95.18) | <0.001 |
| Former-smokers | 1,072 (16.76) | 1,028 (32.50) | 44 ( 1.36) |  |
| Current-smokers | 1,618 (25.29) | 1,506 (47.61) | 112 ( 3.46) |  |
| Drinking status, N (%) |  |  |  |  |
| Never- drinker | 2,846 (44.48) | 566 (17.89) | 2,280 (70.48) | <0.001 |
| Former-drinker | 384 ( 6.00) | 283 ( 8.95) | 101 ( 3.12) |  |
| Current-drinker | 3,168 (49.52) | 2,314 (73.16) | 854 (26.40) |  |
| Body mass index, kg/m^2^ | 24.61 ± 0.0 | 24.34 ± 0.1 | 24.88 ± 0.1 | <0.001 |
| Waist circumference, cm | 82.10 ± 0.1 | 83.51 ± 0.1 | 80.72 ± 0.2 | <0.001 |
| Hip circumference, cm | 94.09 ± 0.1 | 94.14 ± 0.1 | 94.05 ± 0.1 | 0.5311 |
| SBP, mmHg | 120.20 ± 0.2 | 121.24 ± 0.3 | 119.18 ± 0.3 | <0.001 |
| DBP, mmHg | 79.77 ± 0.1 | 81.69± 0.2 | 77.89 ± 0.2 | <0.001 |
| Fasting blood glucose, mg/dL | 87.69 ± 0.3 | 90.72 ± 0.4 | 84.72 ± 0.3 | <0.001 |
| Insulin, µIU/㎖ | 7.50 ± 0.1 | 7.02 ± 0.1 | 7.96 ± 0.1 | <0.001 |
| Total Cholesterol, mg/dl | 192.91 ± 0.5 | 194.11 ± 0.6 | 191.74 ± 0.6 | 0.0082 |
| Triglyceride, mg/dl | 160.59 ± 1.3 | 176.51 ± 2.1 | 145.03 ± 1.5 | <0.001 |
| HDL-Cholesterol, mg/dl | 44.7 ± 0.1 | 43.44± 0.2 | 45.92 ± 0.2 | <0.001 |
| CRP, mg/dL | 0.22 ± 0.0 | 0.23 ± 0.0 | 0.21 ± 0.0 | 0.0796 |
| Creatinine, mg/dL | 0.85 ± 0.0 | 0.96 ± 0.0 | 0.75 ± 0.0 | <0.001 |
| Fvc, L | 3.68 ± 0.0 | 4.30 ± 0.0 | 3.07 ± 0.0 | <0.001 |
| Dietary Intake |  |  |  |  |
| Energy, kcal | 1994.47 ± 7.4 | 2072.43 ± 10.1 | 1918.25 ± 10.7 | <0.001 |
| Protein, g | 65.53 ± 0.3 | 68.59 ± 0.4 | 62.54 ± 0.4 | <0.001 |
| Fat, g | 31.04 ± 0.2 | 34.67 ± 0.3 | 27.49 ± 0.3 | <0.001 |
| Carbohydrate, g | 357.67 ± 1.3 | 364.74 ± 1.7 | 350.75 ± 2.0 | <0.001 |
| Percentages of energy intake of ^4)^ |  |  |  |  |
| Protein, % | 13.04 ± 0.0 | 13.13 ± 0.0 | 12.95 ± 0.0 | 0.0010 |
| Fat, % | 13.53 ± 0.1 | 14.58 ± 0.1 | 12.50 ± 0.1 | <0.001 |
| Carbohydrate, % | 72.23 ± 0.1 | 70.90 ± 0.1 | 73.53 ± 0.1 | <0.001 |

SBP: Systolic Blood Pressure, DBP: Diastolic Blood Pressure, HDL-Cholesterol: High Density Lipoprotein Cholesterol.

1) The P values were calculated using a chi-squared test for categorical variables and a linear regression analysis for continuous variables when comparing the baseline characteristics between men and women. 2) Data are presented as means ± standard deviation (SD) or numbers (%). 3) Household income level was grouped based on quartiles in our study population. 4) Percentages of energy intake from protein, fat and carbohydrate were calculated as follows: protein intake and carbohydrate intake (g/day) X 4kcal/ total energy(kcal/day) X 100%, fat intake (g/day) X 9kcal/ total energy(kcal/day) X100%

**Supplementary Table 3.** Baseline characteristics according to survivors and deaths of participants in the Ansan (Ansan-Ansung) study.

| Characteristics | Total | Survival | Death | P-value^1)^ |
| --- | --- | --- | --- | --- |
| N (%) | 6,398 (100.00)^2)^ | 5,464 (85.40) | 934 (14.60) |  |
| Age (years) | 51.23 ± 0.1^2)^ | 49.85 ± 0.1 | 59.32 ± 0.3 | <0.001 |
| Physiology Healthy Aging Index | 8.96 ± 0.0 | 9.08 ± 0.0 | 8.20 ± 0.0 | <0.001 |
|  | (3.0-10.0) | (4.0-10.0) | (3.0-10.0) |  |
| Education level, N (%) |  |  |  |  |
| Elementary school or below | 1,827 (28.56) | 1,355 (24.80) | 472 (50.54) | <0.001 |
| Middle school | 1,407 (21.99) | 1,213 (22.20) | 194 (20.77) |  |
| High school | 2,162 (33.79) | 1,978 (36.20) | 184 (19.70) |  |
| College or above | 1,002 (15.66) | 918 (16.80) | 84 ( 8.99) |  |
| Household income, N (%) ^3)^ |  |  |  |  |
| Quartile 1 | 1,881 (29.40) | 1,356 (24.82) | 525 (56.21) | <0.001 |
| Quartile 2 | 909 (14.21) | 802 (14.68) | 107 (11.46) |  |
| Quartile 3 | 2,258 (35.29) | 2,041 (37.35) | 217 (23.23) |  |
| Quartile 4 | 1,350 (21.10) | 1,265 (23.15) | 85 ( 9.10) |  |
| Smoking status, N (%) |  |  |  |  |
| Never- smokers | 3,708 (57.96) | 3,294 (60.29) | 414 (44.33) | <0.001 |
| Former-smokers | 1,072 (16.76) | 882 (16.14) | 190 (20.34) |  |
| Current-smokers | 1,618 (25.29) | 1,288 (23.57) | 330 (35.33) |  |
| Drinking status, N (%) |  |  |  |  |
| Never- drinker | 2,846 (44.48) | 2,450 (44.84) | 396 (42.40) | <0.001 |
| Former-drinker | 384 ( 6.00) | 289 ( 5.29) | 95 (10.17) |  |
| Current-drinker | 3,168 (49.52) | 2,725 (49.87) | 443 (47.43) |  |
| Body mass index, kg/m^2^ | 24.61 ± 0.0 | 24.70 ± 0.0 | 24.13 ± 0.1 | <0.001 |
| Waist circumference, cm | 82.10 ± 0.1 | 81.88 ± 0.1 | 83.40 ± 0.3 | <0.001 |
| Hip circumference, cm | 94.09 ± 0.1 | 94.36 ± 0.1 | 92.51 ± 0.2 | <0.001 |
| SBP, mmHg | 120.20 ± 0.2 | 118.76 ± 0.2 | 128.60 ± 0.7 | <0.001 |
| DBP, mmHg | 79.77 ± 0.1 | 79.26 ± 0.2 | 82.77 ± 0.4 | <0.001 |
| Fasting blood glucose, mg/dL | 87.69 ± 0.3 | 86.67 ± 0.3 | 93.61 ± 1.1 | <0.001 |
| Insulin, µIU/㎖ | 7.50 ± 0.1 | 7.52 ± 0.1 | 7.35 ± 0.1 | 0.2353 |
| Total Cholesterol, mg/dl | 192.91 ± 0.5 | 193.45 ± 0.5 | 189.74 ± 1.3 | 0.0058 |
| Triglyceride, mg/dl | 160.59 ± 1.3 | 159.55 ± 1.4 | 166.69 ± 3.3 | 0.0503 |
| HDL-Cholesterol, mg/dl | 44.7 ± 0.1 | 44.63 ± 0.1 | 45.06 ± 0.4 | 0.2698 |
| CRP, mg/dL | 0.22 ± 0.0 | 0.21 ± 0.0 | 0.31 ± 0.0 | <0.001 |
| Creatinine, mg/dL | 0.85 ± 0.0 | 0.85 ± 0.0 | 0.87 ± 0.0 | 0.0489 |
| Fvc, L | 3.68 ± 0.0 | 3.75 ± 0.0 | 3.50 ± 0.0 | <0.001 |
| Dietary Intake |  |  |  |  |
| Energy, kcal | 1994.47 ± 7.4 | 2003.20 ± 7.9 | 1943.39 ± 20.8 | 0.0073 |
| Protein, g | 65.53 ± 0.3 | 66.14 ± 0.3 | 61.98 ± 0.8 | <0.001 |
| Fat, g | 31.04 ± 0.2 | 31.71 ± 0.2 | 27.12 ± 0.6 | <0.001 |
| Carbohydrate, g | 357.67 ± 1.3 | 357.84 ± 1.4 | 356.64 ± 3.7 | 0.7618 |
| Percentages of energy intake of ^4)^ |  |  |  |  |
| Protein, % | 13.04 ± 0.0 | 13.11 ± 0.0 | 12.61 ± 0.1 | <0.001 |
| Fat, % | 13.53 ± 0.1 | 13.79 ± 0.1 | 11.98 ± 0.2 | <0.001 |
| Carbohydrate, % | 72.23 ± 0.1 | 71.92 ± 0.1 | 74.04 ± 0.2 | <0.001 |

SBP: Systolic Blood Pressure, DBP: Diastolic Blood Pressure, HDL-Cholesterol: High Density Lipoprotein Cholesterol.

1) The P values were calculated using a chi-squared test for categorical variables and a linear regression analysis for continuous variables when comparing the baseline characteristics between men and women. 2) Data are presented as means ± standard deviation (SD) or numbers (%). 3) Household income level was grouped based on quartiles in our study population. 4) Percentages of energy intake from protein, fat and carbohydrate were calculated as follows: protein intake and carbohydrate intake (g/day) X 4kcal/ total energy(kcal/day) X 100%, fat intake (g/day) X 9kcal/ total energy(kcal/day) X100%

6,688 participants

Excluded participants with missing Physiology Health Aging Index components (n=625)

N=6,063

Excluded participants with incomplete information on covariates (n=222)

N=5,841

Excluded participants with implausible energy intake (<500 kcal/day or >5000 kcal/day) (n=394)

N=5,447

Excluded participants who did not attend at follow-up survey (n=630)

N=5,221

Excluded participants with cognitive impairment (n=436)

**Final analytic sample:**

**n=5,011 participants**

**(n=6,054)**

**Supplementary Figure 1.** Flow diagram of the study participants with cognitive impairment.

**Supplementary Table 4.** Hazard ratios with 95% confidence intervals (CI) of cognitive impairment incidence by Physiology Healthy Aging Index category

|  | The Physiology Healthy Aging Index | | | |  | |  |
| --- | --- | --- | --- | --- | --- | --- | --- |
| HRs (95% CIs) | Q1 (lowest) | Q2 | Q3 | Q4 (highest) | | P for trend | |
| No. of at risk | 1,272 | 1,080 | 1,314 | 1,345 | |  | |
| Person-years | 14,336 | 13,116 | 16,329 | 17,035 | |  | |
| Incidence rate per 1000 person-years | 21.76 | 15.40 | 11.33 | 6.52 | |  | |
| No. of event | 312 | 202 | 185 | 111 | |  | |
| Crude model | Reference | 0.67 (0.56–0.80) | 0.49 (0.41–0.58) | 0.28 (0.22–0.34) | | <0.001 | |
| Model 1 | Reference | 0.83 (0.70–1.00) | 0.69 (0.57–0.83) | 0.51 (0.40–0.64) | | <0.001 | |
| Model 2 | Reference | 0.90 (0.75–1.08) | 0.75 (0.62–0.90) | 0.65 (0.51–0.82) | | <0.001 | |

Q, Quartile; Q1: Quartile 1 (lowest), Q4: Quartile 4 (highest); HR, hazard ratio; CI, confidence interval.

Crude model was unadjusted.

Model 1 was adjusted for sex and age (years, continuous).

Model 2 was additionally adjusted Education level (Elementary school or below, Middle school, High school, College or above), Household income level (Quartiles), Smoking status (Never smoker, Former smoker, or Current smoker), Drinking status (Never-drinker, Former-drinker, or Current-drinker), Menopause status (Yes or No), body mass index (kg/m2, continuous), and total energy intake (kcal/day, continuous).

The P value for trend was calculated by including the median quintile values of the Physiology Healthy Aging Index as a continuous term in the model.

10,030 participants

Excluded participants with missing Physiology Health Aging Index components (n=552)

N=9,478

Excluded participants with incomplete information on covariates (n=2,414)

N=7,064

Excluded participants with implausible energy intake (<500 kcal/day or >5000 kcal/day) (n=175)

N=6,889

Excluded participants who did not attend at follow-up survey (n=491)

N=6,398

Excluded participants who had hypotension (n=106)

N=6,292

Excluded participants who had hypoglycemia (n=168)

**Final analytic sample:**

**n=6,124 participants**

**(n=6,054)**

**Supplementary Figure 2.** Sensitivity analysis study participants: Flow diagram.

**Supplementary Table 5.** Associations between the physiologically healthy aging index and all-cause, age-related, cancer, and CVD mortality in a sensitivity analysis of study participants

|  | The Physiology Healthy Aging Index | | | |  | |  |
| --- | --- | --- | --- | --- | --- | --- | --- |
| HRs (95% CIs) | Q1 (lowest) | Q2 | Q3 | Q4 (highest) | | P for trend | |
| No. of at risk | 710 | 1,169 | 1,779 | 2,466 | |  | |
| Person-years | 10,890 | 18,547 | 30,006 | 41,629 | |  | |
| Incidence rate per 1000 person-years | 15.3 | 15.9 | 16.9 | 16.9 | |  | |
| All–cause |  |  |  |  | |  | |
| No. of event | 225 | 255 | 214 | 168 | |  | |
| Crude model | Reference | 0.61 (0.51–0.72) | 0.30 (0.25–0.36) | 0.18 (0.14–0.21) | | <0.001 | |
| Model 1 | Reference | 0.83 (0.70–0.99) | 0.50 (0.42–0.61) | 0.52 (0.42–0.65) | | <0.001 | |
| Model 2 | Reference | 0.85 (0.71–1.01) | 0.50 (0.41–0.60) | 0.51 (0.41–0.64) | | <0.001 | |
| Age-related |  |  |  |  | |  | |
| No. of event | 214 | 217 | 181 | 136 | |  | |
| Crude model | Reference | 0.61 (0.51–0.74) | 0.30 (0.24–0.36) | 0.16 (0.13–0.20) | | <0.001 | |
| Model 1 | Reference | 0.85 (0.70–1.02) | 0.51 (0.42–0.62) | 0.52 (0.41–0.66) | | <0.001 | |
| Model 2 | Reference | 0.87 (0.72–1.05) | 0.51 (0.42–0.62) | 0.52 (0.41–0.66) | | <0.001 | |
| Cancer |  |  |  |  | |  | |
| No. of event | 82 | 87 | 84 | 86 | |  | |
| Crude model | Reference | 0.63 (0.47–0.86) | 0.35 (0.26–0.48) | 0.26 (0.19–0.36) | | <0.001 | |
| Model 1 | Reference | 0.80 (0.59–1.08) | 0.54 (0.40–0.74) | 0.69 (0.49–0.96) | | 0.0035 | |
| Model 2 | Reference | 0.81 (0.60–1.10) | 0.53 (0.39–0.73) | 0.67 (0.48–0.94) | | 0.0021 | |
| CVD |  |  |  |  | |  | |
| No. of event | 63 | 66 | 32 | 15 | |  | |
| Crude model | Reference | 0.59 (0.42–0.83) | 0.16 (0.11–0.25) | 0.06 (0.03–0.10) | | <0.001 | |
| Model 1 | Reference | 0.93 (0.65–1.31) | 0.32 (0.21–0.50) | 0.24 (0.13–0.44) | | <0.001 | |
| Model 2 | Reference | 0.94 (0.66–1.34) | 0.33 (0.22–0.51) | 0.26 (0.14–0.47) | | <0.001 | |

Q, Quartile; Q1: Quartile 1 (lowest), Q4: Quartile 4 (highest); CVD, cardiovascular disease; HR, hazard ratio; CI, confidence interval.

Crude model was unadjusted.

Model 1 was adjusted for sex and age (years, continuous).

Model 2 was additionally adjusted Education level (Elementary school or below, Middle school, High school, College or above), Household income level (Quartiles), Smoking status (Never smoker, Former smoker, or Current smoker), Drinking status (Never-drinker, Former-drinker, or Current-drinker), Menopause status (Yes or No), body mass index (kg/m2, continuous), and total energy intake (kcal/day, continuous).

The P value for trend was calculated by including the median quintile values of The Physiology Healthy Aging Index as a continuous term in the model.
